# Supplementary figures and images for: Pigment analysis based on a line-scanning fluorescence hyperspectral imaging microscope combined with multivariate curve resolution
Source: PLoS One. 2021 Aug 9;16(8):e0254864. doi: 10.1371/journal.pone.0254864 (PMC8351980; doi:10.1371/journal.pone.0254864)

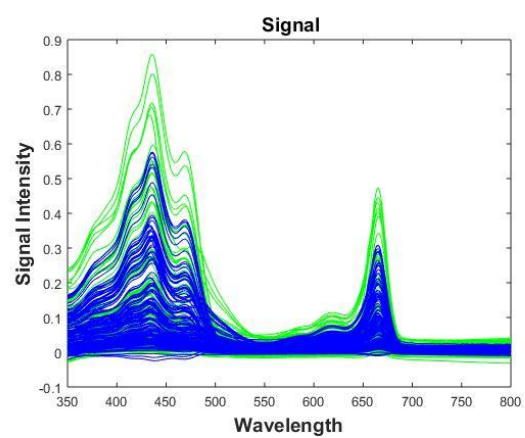

**S1 Fig Absorption spectrum of 147 samples**

Supplement: S1 Fig — (PDF) [file pone.0254864.s001.pdf]

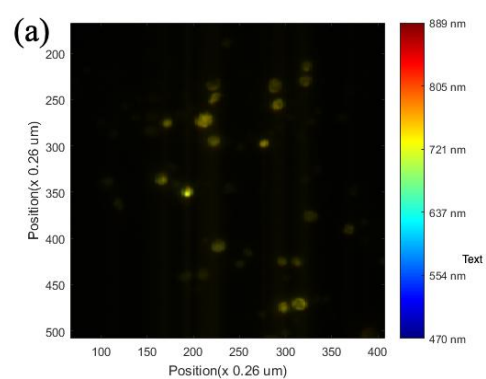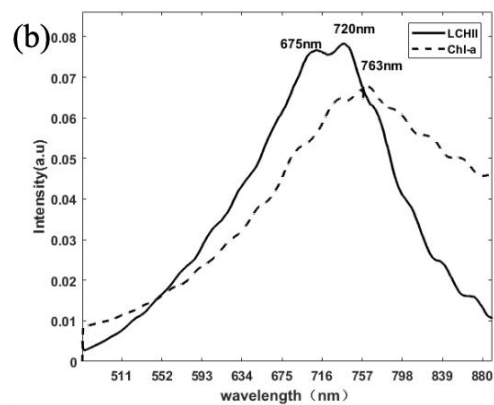

**S2 figure Chlorella sp of N-normal condition (a)fusion image (b)pure component spectrum**

Supplement: S2 Fig — (a)fusion image (b)pure component spectrum. (PDF) [file pone.0254864.s002.pdf]
