# Supplementary material for: Pigment analysis based on a line-scanning fluorescence hyperspectral imaging microscope combined with multivariate curve resolution
Source: PLoS One. 2021 Aug 9;16(8):e0254864. doi: 10.1371/journal.pone.0254864 (PMC8351980; doi:10.1371/journal.pone.0254864)
Supplement: S3 Fig — (a) overlapped and concentrated cells (b) sparse cells. (PDF) [file pone.0254864.s003.pdf]

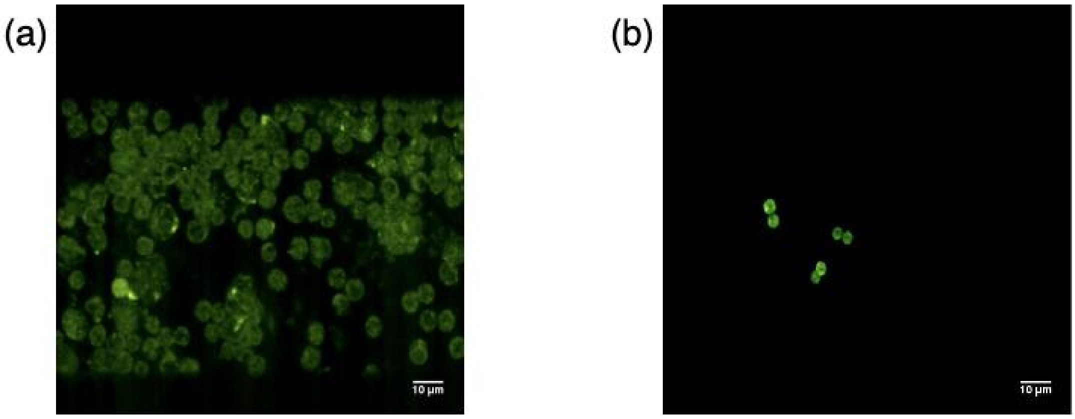

S3 figure The images of Cyanobacterial algae under different concentration (a) overlapped and concentrated cells (b) sparse cells
