# Supplementary material for: Pigment analysis based on a line-scanning fluorescence hyperspectral imaging microscope combined with multivariate curve resolution
Source: PLoS One. 2021 Aug 9;16(8):e0254864. doi: 10.1371/journal.pone.0254864 (PMC8351980; doi:10.1371/journal.pone.0254864)
Supplement: S1 Table — (PDF) [file pone.0254864.s004.pdf]

S1 Table System parameter

	Spectrometer			CCD Camera		Motored Stage	
Quantity	Slit (μm)	Grating (lpmm)	Spectrum range (nm)	Size (pixels)	Pixel size (μm/pixel)	Accuracy (μm)	Stage size (mm)
Value	10-3000	150/1200	450-800	1024*1024	13	0.05	295(L)*260(W)*32.5(H)
