# Supplementary material for: Pigment analysis based on a line-scanning fluorescence hyperspectral imaging microscope combined with multivariate curve resolution
Source: PLoS One. 2021 Aug 9;16(8):e0254864. doi: 10.1371/journal.pone.0254864 (PMC8351980; doi:10.1371/journal.pone.0254864)
Supplement: S2 Table — (PDF) [file pone.0254864.s005.pdf]

S2 Table The concentration of pigment

Component	CHl		PC		APC		CAROTENOID	
**Content**	C (mg/ml)	Percentage (%)	C (mg/ml)	Percentage (%)	C (mg/ml)	Percentage (%)	C (mg/ml)	Percentage (%)
**Anabaena**	6.67E-3	0.84	7.71E-1	95.51	2.95E-2	3.65	0	0
**Chlorella sp.**	1.53E-2	99.75	0	0	0	0	3.84E-5	0.25
**Microcytic aeruginosa**	2.8E-3	9.30	2.25E-2	74.70	4.82E-3	16.00	0	0

[a]C denotes relative concentration, it isn't a accurate value.

[b]CHI is the aberration of chlorophyll, PC is phycocyanin, APC is allophycocyanin.
