# Supplementary material for: Pigment analysis based on a line-scanning fluorescence hyperspectral imaging microscope combined with multivariate curve resolution
Source: PLoS One. 2021 Aug 9;16(8):e0254864. doi: 10.1371/journal.pone.0254864 (PMC8351980; doi:10.1371/journal.pone.0254864)
Supplement: Attachment — Submitted filename: Response to Reviewers.docx [file pone.0254864.s006.docx]

Response to Reviewers1:

Reviewer #1: 

1. “Another approach is to capture images based on specific fluorescence signatures [3]. That means that the value of fluorescent peaks is considered as the gold standard for pigment analysis” (lines 33 and 34). What does the gold standard mean? and is it another approach HSI?

Answer Q1:

Another approach in this sentence(lines 33 and 34) is exactly the specialized microscope. The manuscript presents the hyperspectral imaging microscope based on the line-scanning method. The method acquires the spatial and spectral information and reconstructs the image according to the fluorescent feature. Therefore another approach in the paper is based on the fluorescent feature to catch the images of samples.

2 The author argued that ‘HSI is easy to implement, fast and reliable (line 68). Why?

Answer Q2:

Hyperspectral imaging microscope is a method with the advantages of acquiring spatial and spectral information simultaneously. The system contains two parts: the hardware and the software. We programmed the user interface to control the movement of stage and capture of images. It is concise and easily operated. As soon as just pressing the button, we acquire the hyperspectral images of samples. Moreover, the system is based on the line scanning, that is, scanning the sample each time we can get spectral information of a whole line. It costs time shorter than the point-scanning method.

3. Sometimes, the authors use the abbreviation without definition.

Answer Q3:

From the whole manuscript, I found the lines 60 and 99 has a abbreviation without definition. The GFP is the abbreviation of green fluorescent protein; the MCR-ALS algorithm is the abbreviation of multivariate curve resolution - alternating least squires. The MCR-ALS algorithm develops based on the MCR algorithm.

4. In line 189 ‘Spectroscopy contains two gratings with a blaze wavelength of 600 nm and grooves of 300 mm-1 and 1200 mm-1’, the unit of grating is not correct.

Answer Q4:

The unit of grating isn't mm^-1^ but lpmm, which is the abbreviation of line pairs per mini-meter. It means that the grating has two gratings. One is 300 line pairs per mini-meter in the surface. Anther is 1200 line pairs per mini-meter.

Response to Reviewers2:

Reviewer #2:

1. the line 176 ‘For a very low algae concentration, the fluorescence intensity was linearly related to the concentration of the fluorescent substance’ is the basic principle to calculate the content of pigments. However, the value of low algae concentration could not be found from the whole manuscript, and it should be given in revised manuscript.

Answer Q1:

Before the experiment, we diluted the samples with the original solution to culture medium at a ratio of 1: 1000 in the sample preparation. The sample has more than the original concentration of 10e6 cells per milliliter. Moreover, in order to verify the limited numbers of microalgal cells we can catch the images of algae under different concentration. It concludes that the detection limited is more than two cells has enough intensity that could be detected. The image in S3 figure is full of cells, which isn’t classified for several cells. What‘s more, we catch the image without overlapping cells that can be distinguished.

2. The description and presentation of results aren’t clear. For example, ‘For this work, hyperspectral imaging combined with the MCR algorithm simultaneously got the spectral features and pigment location. The spectral features of the pigment components are consistent with the fluorescence excitation spectrum in the verification experiment.’ It is also not clear for the data of verification experiment. Please make description in detail. (lines 338 and 339).

Answer to Q2:

In the verification experiment, we have got the data of the absorption spectrum and the emission spectrum using 95% alcohol to extracting the pigment in algae. Emission measurement excited at 512 nm (ex: 512 nm) after pigment extraction. The peaks of emission spectrum are at 671 nm, 587 nm, 492 nm and 438 nm(figure 8), which is consistent with the peaks of 675nm and sub-peak ranges from 470nm to 574nm respectively in the hyperspectral images(Fig S2). The fluorescent excitation spectrum of Chlorella sp. indicates that the pigment composition is Chl-a (671 nm) and carotenoid (492 nm). Therefore，the spectral feature of the pigment components are consistent with the fluorescence excitation spectrum.

3. Some expressions should be refined by native English speakers.

Answer to Q3:

The manuscript is revised through native speaker in spelling, English-grammar and language usage. I employed the professional scientific editing service to check the the text.

Thanks for your good comments.
